# Mouse Genetic Models Reveal Surprising Functions of IκB Kinase Alpha in Skin Development and Skin Carcinogenesis

**DOI:** 10.3390/cancers5010170

**Published:** 2013-02-15

**Authors:** Xiaojun Xia, Eunmi Park, Susan M. Fischer, Yinling Hu

**Affiliations:** 1 The Methodist Hospital Research Institute, Houston, TX 77030, USA; 2 Department of Radiation Oncology, Dana-Farber Cancer Institute, Harvard Medical School, Boston, MA 02115, USA; 3 Department of Molecular Carcinogenesis, The University of Texas MD Anderson Cancer Center, Smithville, TX 78967, USA; 4 Cancer and Inflammation Program, Center for Cancer Research, Frederick National Laboratory for Cancer Research, Frederick, MD 21701, USA

**Keywords:** skin carcinogenesis, skin development, IKKalpha (IKKα)

## Abstract

Gene knockout studies unexpectedly reveal a pivotal role for IκB kinase alpha (IKKα) in mouse embryonic skin development. Skin carcinogenesis experiments show that *Ikkα* heterozygous mice are highly susceptible to chemical carcinogen or ultraviolet B light (UVB) induced benign and malignant skin tumors in comparison to wild-type mice. IKKα deletion mediated by keratin 5 (K5).Cre or K15.Cre in keratinocytes induces epidermal hyperplasia and spontaneous skin squamous cell carcinomas (SCCs) in *Ikkα* floxed mice. On the other hand, transgenic mice overexpressing IKKα in the epidermis, under the control of a truncated loricrin promoter or K5 promoter, develop normal skin and show no defects in the formation of the epidermis and other epithelial organs, and the transgenic IKKα represses chemical carcinogen or UVB induced skin carcinogenesis. Moreover, IKKα deletion mediated by a mutation, which generates a stop codon in the *Ikkα* gene, has been reported in a human autosomal recessive lethal syndrome. Downregulated IKKα and *Ikkα* mutations and deletions are found in human skin SCCs. The collective evidence not only highlights the importance of IKKα in skin development, maintaining skin homeostasis, and preventing skin carcinogenesis, but also demonstrates that mouse models are extremely valuable tools for revealing the mechanisms underlying these biological events, leading our studies from bench side to bedside.

## 1. Introduction

IκB kinase alpha (IKKα), also known as CHUK, is an 85-kD polypeptide with 745 amino acids (aa). It consists of a putative kinase catalytic domain (KD, aa 15–300) containing 12 regions of homology that are characteristic of a protein serine kinase, a leucine zipper (LZ) motif, and a helix-loop-helix (HLH) motif, and it belongs to a classic zipper protein family, which includes c-myc, Id1, C/EBP, and Jun [1,2]. IKKα can form homodimers and heterodimers, which lead to distinct pathways in a cell-specific manner *in vivo* [3,4]. For example, IKKα, IKKβ, and IKKγ (NEMO) form an IKK complex, which is essential for activating the canonical NF-κB pathway. IKKα and IKKβ are two highly conserved protein kinases, and IKKγ is a regulatory subunit. On the other hand, IKKα homodimers lead to noncanonical NF-κB signaling. Unlike IKKβ and IKKγ, mouse genetic studies unexpectedly disclose that IKKα deletion impairs embryonic skin development, and that IKKα homodimers show transcription activity in regulating the expression of many genes encoding proteins that are important for skin and skin tumor development [5,6,7,8]. In this review, we discuss how different mouse models illustrate the physiological functions of IKKα in the skin.

## 2. Why Is It Surprising that IKKα Has a Role in Embryonic Skin Development?

IKKα is identified as the first component of the IKK complex, which activates NF-κB, following tumor necrosis factor (TNF), interleukin 1 (IL-1), or lipopolysaccharide (LPS) stimulation, through phosphorylating IκBα, an NF-κB inhibitor, thereby inducing IκBα ubiquitination and degradation in cell lines [4,9,10]. NF-κB has an anti-apoptotic activity via the TNF receptor (TNFR) death pathway [4]. Mice lacking p65 (RelA, a major NF-κB subunit in the canonical NF-κB pathway), IKKβ, or IKKγ die at days 15.5, 13.5, and 12.5, respectively, during embryonic development due to liver cell apoptosis-induced hemorrhage [11,12,13]. Depleting TNFR rescues *p65^−/−^*, *Ikkβ^−/−^*, and *Ikkγ^−/−^* mice [14,15,16,17,18,19], indicating that p65, IKKβ, and IKKγ antagonize apoptosis downstream of the TNFR-mediated death pathway. In addition, *p65^−/−^/Tnfr^−/−^*, *Ikkβ^−/−^/Tnfr^−/−^*, and *Ikkγ^−/−^/Tnfr^−/−^* newborn mice develop relatively normal skin. No severe skin lesions have been reported in mice lacking one of the other NF-κB genes [4]. Most NF-κB family members function in the development of lymphoid cells and lymphoid organs [4]. Because IKKβ has stronger kinase activity than IKKα, and IKKα and IKKβ share similar protein motifs and can phosphorylate the same proteins [20], it was speculated that IKKα and IKKβ were functionally redundant *in vivo* and that IKKα knockout might not have phenotypes due to the presence of IKKβ and IKKγ.

Surprisingly, *Ikkα^−/−^* newborn mice exhibit the appearance of a pupa with shiny, wrinkleless skin, and these newborns die soon after birth because of quick loss of body water [15,21,22]. Histological examination and immunostaining show that the *Ikkα^−/−^* epidermis is markedly thick compared to wild-type (WT), and that the entire *Ikkα^−/−^* epidermis expresses the basal epidermal cell markers keratin 5 (K5) and K14 and lacks the determinably differentiated cell layers in the epidermis that blocks body water loss. Because of no cornfield layers formed on top of the *Ikkα^−/−^* skin, the epidermis between the limbs and body begins fuse together after day 13 during *Ikkα^−/−^* mouse embryonic development. At birth, the invisible limbs are developing beneath the inner skin of *Ikkα^−/−^* mice [21]. These phenotypes demonstrate that IKKα is required for epidermis formation during embryonic development. In the skin, the basal keratinocytes are able to proliferate [23]. Once keratinocytes move to the superficial layer, they stop proliferating and start to differentiate. Those normal differentiating keratinocytes are no longer able to express basal keratinocyte markers, such as K5 and K14. IKKα loss cannot turn off the expression of K5 and K14, and K5 and K14 are co-expressed with differentiating keratinocyte markers K1, K10, and involucrin in the superficial epidermal layers of *Ikkα^−/−^* skin. It is still unknown how IKKα loss interrupts the expression patterns of these distinct keratins during the formation of the epidermis. Importantly, NF-κB activity is not reduced in *Ikkα^−/−^* keratinocytes and depleting TNFR does not alter the skin phenotypes in *Ikkα^−/−^*/*Tnfr^−/−^* mice [15,21,24]. Thus, it is clear that *Ikkα^−/−^* skin phenotypes are not directly caused by a defect in the activation of IKKβ, IKKγ, p65, and TNFR pathway.

Interestingly, *interferon regulator factor 6* (IRF6) knockout and repeated epilation (*Er/Er*) newborn mice display a skin phenotype and appearance similar to that of *Ikkα^−/−^* newborn mice [25,26,27]. IRF6 is a member of the IRF transcription factor family (IRF1-IRF9). Mutations in Irf6 have been reported to cause Van der Woude and popliteal pterygium syndromes, which result in the facial development of cleft lips and palates in humans [28]. IKKα and IRF6 knockout mice display similar phenotypes in facial and tooth development [29]. In *Er/Er* mice, a mutation is found in *14-3-3σ*, which generates an unstable and truncated 14-3-3σ protein [26,27]. 14-3-3σ functions as a G2/M cell-cycle checkpoint in response to DNA damage [30,31]. Although *Ikkα^−/−^*, *Irf6^−/−^*, and *Er/Er* newborn mice exhibit shiny and thick skin with a similar appearance, IKKα expression levels are comparable in *Irf6^−/−^*, *Er/Er*, and WT mice, suggesting that either IKKα, IRF6, and 14-3-3σ lead to different pathways but crosstalk, or that IKKα is an upstream regulator of IRF6 and 14-3-3σ. We have reported that IKKα upregulates 14-3-3σ expression at the transcriptional level in an epigenetic manner [31]. Loss of IKKα does not completely block 14-3-3σ expression and other molecules also regulate the expression of 14-3-3σ [32]. Interestingly, our study found that when using the same culture conditions, *Ikkα^−/−^* and *Er/Er* keratinocytes do not undergo terminal differentiation, but *Ikkα^−/−^* keratinocytes can grow into large colonies compared to *Er/Er* keratinocytes [33], suggesting that IKKα and 14-3-3σ may lead to different signaling pathways in regulating keratinocyte growth.

It has been speculated for a long time that IKKα and p63 crosstalk because *Ikkα^−/−^* and *p63^−/−^* mice show opposite skin phenotypes [21,34]. *p63^−/−^* mice die at birth and have an extremely thin epidermis compared to WT mice. Several studies have identified IKKα as a downstream target of p63 in epidermis formation [35,36,37]. Furthermore, IKKα has been shown to regulate the expression of Mad1, Mad2, and Ovol1 in keratinocyte proliferation and differentiation [6,7,38]. Because the defect in the embryonic skin development of *Ikkα^−/−^* mice has not been rescued by any of those genes described above, the genetic pathways IKKα-mediated in regulating embryonic development remain to be disclosed.

Importantly, IKKα deficiency is associated with a lethal inherited human disease (called cocoon syndrome) [39]. The human fetuses at 13 to 14 gestational weeks display multiple severe malformations in the cranial and craniofacial area and their appearance is similar to *Ikkα^−/−^* knockout embryos [21,40]. The defects in multiple organs including the kidney, lungs, skeletal muscles, diaphragm, and limbs and increased vascularization in the paws and omphalocele were also observed. These lethal fetuses remain normal karyotypes, but contain a mutation from C→T at the nucleotide 1,264 in the *Ikkα* gene, which generates a stop codon at position 422 of IKKα protein, leading to a truncated polypeptide of 421 amino acids of IKKα. Previously, we demonstrated that the truncated IKKα proteins were unstable and had no functions in phosphorylating proteins and inducing keratinocyte differentiation [24]. Because these human fetuses die before the keratinization of the epidermis, no defects in the skin development are detected. Consistently, *Ikkα^−/−^* mouse embryos display epidermal hyperplasia after E16 to E17 days. These results indicate that IKKα is critical for human development.

## 3. Loss and Gain of IKKα Function in Skin Carcinogenesis

Chemical carcinogen and ultraviolet B (UVB) induced skin carcinogenesis approaches have been widely used to study the mechanisms of skin carcinogenesis, to reveal the unknown activity of molecules, and to identify therapeutic reagents to treat and prevent skin tumors [41,42,43,44,45]. These two approaches target different pathways. For example, the chemical carcinogen 7,12-dimethylbenz[a]anthracene (DMBA) induces activating mutations in *H-ras*, and the tumor promoter 12-*O*-tetradecanoylphorbol-13-acetate (TPA), an inflammation irritator, expands H-Ras-targeted cells and elicits skin inflammation. On the other hand, UVB causes DNA damage and *p53* mutations in the skin. Both skin carcinogenesis models require inflammation to promote skin tumor development [41,46].

### 3.1. Chemical Carcinogen-Induced Skin Carcinogenesis

Because *Ikkα^−/−^* mice die soon after birth, we studied the activity of IKKα in skin tumor development in *Ikkα^+/−^* mice on a C57/BL6 background using DMBA and TPA treatment [41]. *Ikkα^+/−^* mice develop two times more benign skin papillomas and 11 times more malignant carcinomas, resembling human squamous cell carcinomas (SCCs), compared to WT mice. The tumor latency is shorter and the tumor sizes are larger in *Ikkα^+/−^* than in WT mice. Importantly, the WT *Ikkα* allele is lost in half of the *Ikkα^+/−^* papillomas and in almost all of the *Ikkα^+/−^* carcinomas. We also observed undifferentiated features in skin carcinomas lacking IKKα compared to carcinomas having IKKα. These results demonstrate that *Ikkα* is haploid insufficient for suppressing tumor progression, that somatic IKKα deletion can facilitate skin carcinoma development, and that the tumor progression may be related to a de-differentiation program in keratinocytes. Furthermore, we found a similar rate of *H-ras* mutations in both *Ikkα^+/−^* and WT skin tumors, while *Ikkα^+/−^* mice developed more skin tumors than WT mice, suggesting that, at the tumor initiation stage, IKKα reduction promotes H-Ras-targeted cell expansion following TPA treatment, which accelerates skin tumor formation. In addition, we also identified mutations in the *Ikkα* gene in WT and *Ikkα^+/−^* skin papillomas and carcinomas. *Ikkα* mutations in *Ikkα^+/−^* papillomas may trigger *Ikkα* WT allele loss, thus promoting carcinoma development (tumor progression) [41]. These results also suggest that *Ikkα* is a target of mutagenesis during skin carcinogenesis. Following TPA treatment, we observed the increased expression of multiple growth factors, cytokines, and vascular factors, and increased extracellular signal-regulated kinase (ERK) activity in *Ikkα^+/−^* skin compared to WT skin [41], all of which further support that IKKα reduction amplifies the Ras and mitogenic pathways and elevates micro-blood vessel formation. Collectively, these results indicate that IKKα is a tumor suppressor of skin SCCs.

On the other hand, IKKα overexpression in the epidermis represses tumor progression and metastases in Lori.IKKα transgenic mice on a FVB background compared to WT mice in DMBA/TPA- and DMBA/DMBA-induced skin carcinogenesis settings [47]. Transgenic IKKα inhibits DMBA/TPA-induced mitogenic and angiogenic activities. The study on gain of IKKα function further supports that IKKα acts as a tumor suppressor in skin carcinoma development [47].

### 3.2. UVB-Induced Skin Carcinogenesis

UVB is an etiological cause of human skin cancer, and the tumor suppressor p53 is one of its targets [44,48]. UVB induces p53 mutations in the skin at the early stage of skin carcinogenesis and causes skin carcinomas, resembling human SCCs, in hairless mice [49,50]. On the other hand, UVB-induced DNA damage mediates cell death, which serves as a defense mechanism to clear the cells with DNA damage, thereby preventing the expansion of cells carrying DNA mutations [45]. The development of skin tumors requires repeated treatments of UVB. Increased infiltrating inflammatory cells in the skin following UVB treatment are a common phenomenon, and inflammation is pivotal for UVB skin carcinogenesis. UVB treatment induces more p53 mutations and many more skin carcinomas, and shorter tumor latency in *Ikkα^+/−^* than in WT hairless mice [49]. Cell death numbers are significantly reduced in UVB-treated *Ikkα^+/−^* skin compared to UVB-treated WT skin. Furthermore, we observed increased cytokines, including TNF, IL-1, IL-6, and monocyte chemoattractant protein-1 (MCP-1), and increased macrophages in the skin of UVB-treated *Ikkα^+/−^* mice compared to WT mice. Therefore, IKKα reduction promotes DNA damage and inflammation but decreases apoptosis. NF-κB activity is higher in *Ikkα^+/−^* than in WT keratinocytes following TNF treatment, which may contribute into increased inflammation and reduced apoptosis. Likely, this increased NF-κB is a result of increased IKK activity as IKKβ replacing IKKα in the IKK complex has stronger kinase activity [24]. Although increased p53 mutations and NF-κB activity may contribute to decreased cell death following UVB treatment in *Ikkα^+/−^* skin compared to WT skin, there are likely additional mechanisms to be revealed. 

**Figure 1:**
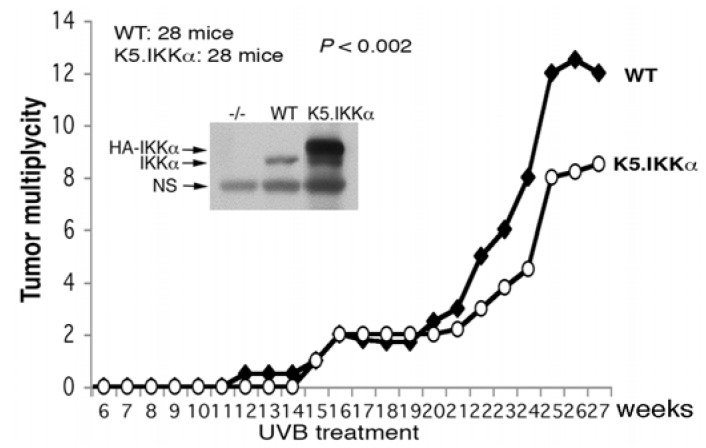
Tumor multiplicity in UVB-treated wild-type (WT) and K5.IKKα transgenic mice. Each group contains 28 mice in SKH-1 background. Western blot shows IKKα levels in the skin of *Ikkα^−/−^*, WT, and K5.IKKα mice. NS, a non-specific band; HA, hemagglutinin-A tag. UVB treatment was described as in reference [49].

On the other hand, K5.IKKα transgenic mice develop normal skin, do not show any histological abnormalities, and rescue the skin phenotype of *Ikkα^−/−^* mice in an IKKα dose-dependent manner [38]. The transgenic IKKα has been shown to repress IKKα loss-induced the activities of epidermal growth factor receptor (EGFR), ERK, c-Jun N-terminal kinase (JNK), activator protein 1 (AP-1), and signal transducer and activator of transcription 3 (Stat3), and to elevate the expression of Ovol1 and Mad1, which are c-myc antagonists, in the skin of *Ikkα^−/−^*/K5.IKKα mice [38]. Furthermore, the K5.IKKα transgene significantly inhibits UVB-induced skin tumor development in K5.IKKα mice compared to WT mice (see Figure 1). Lori.IKKα also inhibits UVB-induced skin carcinogenesis (data not shown). Together, these results demonstrate that IKKα is important for suppressing UVB-induced skin carcinogenesis. Both chemical and UVB skin carcinogenesis models demonstrate that increased IKKα has an inhibitory effect on skin carcinogenesis; thus, IKKα may be considered a target for preventing skin tumor development.

Although newborn mice lacking IKKα, 14-3-3σ, or IRF6 display similar skin appearance, there is no direct evidence that these molecules function within the same genetic pathway. Interestingly, *Er/+* mice are more susceptible to DMBA/TPA-induced skin carcinogenesis than WT mice [51]. *Er/+/p63^+/−^* mice developed fewer skin tumors than *Er/+* mice, although the tumor incidence in *p63^+/−^* and WT mice was similar, suggesting that reduced p63 antagonizes 14-3-3σ reduction-promoted skin tumor development. In addition, IRF6 is downregulated in human skin SCCs, and has a tumor suppressive function [52,53]. These findings suggest that the developmental-phenotypic-related genes of IKKα, 14-3-3σ, and IRF6 are tumor suppressive. Whether the deletion of 14-3-3σ or IRF6 also induces spontaneous skin SCCs in a manner similar to IKKα remains to be determined [5].

## 4. Keratinocyte-Specific IKKα Deletion Induces Epidermal Hyperplasia and Spontaneous Skin SCCs; EGFR Reduction, but not TNFR Loss, Inhibits IKKα Loss-Mediated Epidermal Hyperplasia

TNFR deletion rescues *Ikkβ^−/−^* and *Ikkγ^−/−^* mice. *Ikkβ^−/−^/Tnfr^−/−^* and *Ikkγ^−/−^/Tnfr^−/−^* mice show no defects in skin development, whereas TNFR deletion does not rescue the skin phenotype of *Ikkα^−/−^* mice [15]. Thus, IKKβ and IKKγ are not required for mouse embryonic skin development. It is known that IKKα, IKKβ, and IKKγ form the IKK complex, but how can we dissect their physiological functions? Conditional gene deletion in specific cell types in mice may help to address this issue. K14.Cre-mediated deletion of IKKβ or IKKγ specifically in keratinocytes induces epidermal hyperplasia and skin inflammation, and these *Ikkβ^f/f^*/K14.Cre and *Ikkγ^f/f^*/K14.Cre mice die within three weeks after birth [54,55]. Again, TNFR depletion, but not T-cell depletion, rescues skin phenotypes in *Ikkβ^f/f^/K14.Cre/Tnfr^−/−^* and *Ikkγ^f/f^/K14.Cre/Tnfr^−/−^* mice. Inducible K14.CreER-mediated IKKβ, or IKKγ deletion in keratinocytes also causes epidermal hyperplasia and skin inflammation, but not skin tumors, and TNFR deletion, indeed, rescues the skin phenotypes in these mice. Collectively, these results suggest that the IKKβ and IKKγ deletion-mediated skin phenotypes are associated with a TNFR-mediated pathway. While it is known that *Tnfr^−/−^* mice develop fewer skin tumors than WT mice, following DMBA and TPA treatment [56], the effect of IKKβ or IKKγ deletion on skin tumor development remains unknown.

We have shown that paternal K5.Cre- or K14.Cre-mediated IKKα deletion in keratinocytes induces epidermal hyperplasia; *Ikkα^f/f^/K5.Cre* and *Ikkα^f/f^/K14.Cre* mice die within three weeks after birth; and maternal K5.Cre or K14.Cre-mediated IKKα deletion induces the same phenotype as that of *Ikkα^−/−^* mice because K5.Cre and K14.Cre can be activated in oocytes [5,57,58]. Interestingly, TNFR deletion does not rescue these skin phenotypes, but instead slightly enhances the skin phenotypes in *Ikkα^f/f^/K5.Cre* (paternal Cre) mice. In another study [59], Gareus *et al*. used K14.Cre to delete IKKα in *Ikkα^f/f^* mice. Since they did not discuss whether they used paternal or maternal K14.Cre to delete IKKα in the mutant mice, when IKKα was specifically deleted in the epidermis during embryonic development, and whether IKKα was indeed expressed in the dermis and other multiple organs, it is premature to evaluate those results before obtaining the additional information. Furthermore, inducible IKKα deletion in keratinocytes induces spontaneous skin carcinomas in *Ikkα^f/f^/K5.CreER* and *Ikkα^f/f^/K5.CrePR* mice. Cultured IKKα-null keratinocytes obtained from both *Ikkα^f/f^/K5.Cre* and *Ikkα^−/−^* mice exhibit similar undifferentiated and hyperproliferative features [5,24]. Many pathways, including EGFR/H-Ras/ERK signaling, are altered in IKKα-null keratinocytes compared with WT keratinocytes. Inhibiting EGFR, H-Ras, or ERK activity represses keratinocyte proliferation and induces terminal differentiation in IKKα-null keratinocytes in culture. Importantly, using genetic and pharmacological approaches, inhibiting EGFR represses epidermal hyperplasia and prevents spontaneous skin tumors in *Ikkα^f/f^/K5.CreER* mice [5]. In addition, reintroducing IKKα inhibits the expression of genes that encode growth factors and growth factor activators, which serve as EGFR ligands, at the gene transcription levels. Thus, IKKα loss elevates the EGFR activity, enhancing EGFR/H-Ras/ERK activity and the crosstalk between the EGFR pathway and IKKα occurs in the nucleus of keratinocytes [5]. This conclusion may also explain why TNFR depletion does not affect the skin condition of mice lacking IKKα in keratinocytes.

Furthermore, we have shown that IKKα loss-mediated EGFR activity forms a loop with increased Stat3 and AP-1 activities in the skin [38]. Reducing EGFR or reintroducing IKKα can inhibit this autocrine loop, as well as epidermal hyperplasia and skin inflammation including increased infiltrating macrophages and cytokines in *Ikkα^f/f^/K5.Cre* mice. Because TNFR depletion does not rescue the skin phenotypes in *Ikkα^f/f^/K5.Cre/Tnfr^−/−^* mice [5], skin inflammation may not be associated with the TNFR pathway. Increased Stat3 or AP-1 transcription factors also lead to major inflammation pathways [60,61], and, thus, may provide the induction signaling in skin inflammation in *Ikkα^f/f^/K5.Cre* mice. These targets may be considered therapeutic ones for treating and preventing human skin diseases. 

## 5. IKKα Regulates the Cell Cycle in Keratinocytes

The cell cycle regulates keratinocyte proliferation and differentiation. Increased BrdU incorporation is observed in the *Ikkα^−/−^* epidermis compared to WT, but overexpression of p21 and p27 cell-cycle inhibitors is not able to drive *Ikkα^−/−^* keratinocytes to terminal differentiation [21,33], suggesting that the S phase may be an important point that regulates IKKα-null keratinocyte proliferation and differentiation. To support this point, we and others have shown that reintroduced domain-negative H-Ras-N17 represses proliferation, induces terminal differentiation, and inhibits EGFR, ERK, and Ras activities in *Ikkα^−/−^* keratinocytes; increased levels of c-myc antagonists Ovol1 and Mad1 also reverse the status of proliferation and differentiation in *Ikkα^−/−^* keratinocytes through the transforming growth factor beta (TGFβ) and Smad pathway [5,6,7]. TGFβ regulates the c-myc/Ovol1/Mad1 pathway [6]. Thus, both EGFR/H-Ras/ERK and TGFβ/c-myc pathways are crucial for keratinocyte proliferation and differentiation.

We also demonstrated that IKKα prevents trimethylation on the *14-3-3σ* gene promoter by interacting with histone H3 (H3), which further blocks trimethyltransferase (Suv39h1) access to *14-3-3σ* [31]. As mentioned above, 14-3-3σ is a G2/M cell-cycle checkpoint [30]. *Ikkα^−/−^* keratinocytes exhibit a reduction at the G2/M phase. DNA damage increases the G2/M phase in WT keratinocytes, but does not significantly increase the G2/M phase in *Ikkα^−/−^* keratinocytes. Reintroducing IKKα or 14-3-3σ rescues this defect. The G2/M cell-cycle checkpoint function is important for protecting cells from DNA damage. *Ikkα^−/−^* keratinocytes lack the ability to arise the G2/M checkpoint [31]. Therefore, these IKKα-null keratinocytes may be prone to DNA damage.

**Figure 2:**
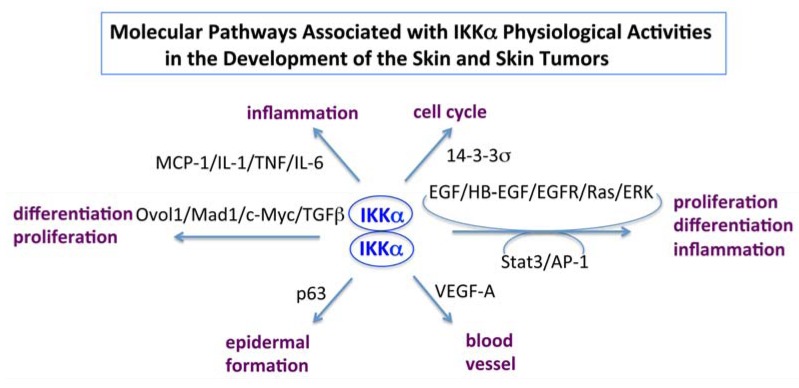
Identified pathways associated with IKKα functions in the skin and skin tumor development in mice. Arrows indicate IKKα-involved.

Taken together, we summarize the molecular pathways associated with IKKα physiological activities in the development of the skin and skin tumors in Figure 2. The most results were obtained from our published studies from 1999 to now. Thus, this review may not include all the published data and we apologize for this.

## 6. Conclusions and Puzzles

It has been puzzling that IKKα and IKKβ display distinct physiological functions although the two protein kinases share similar protein motifs and can phosphorylate the same proteins. For example, K5.IKKβ transgenic mice develop skin inflammation and epidermal hyperplasia [62], but K5.IKKα transgenic mice show no pathological changes in the skin and other organs [38,63]. Depleting TNFR rescues the skin phenotype of *Ikkβ^−/− ^*and *Ikkβ^f/f^*/K14.Cre mice, but does not rescue the skin phenotype of *Ikkα^−/−^* and *Ikkα^f/f^*/K5.Cre mice. It is known that IKKα regulates classical and non-classical NF-κB pathways by forming the IKK complex and IKKα homodimers [4]. The activation of these pathways largely depends on cell receptors. Noncanonical NF-κB is specifically activated in B cells and some stromal cells [64]. We recently showed that in B cells, IKKα inactivation causes increased p100, a target of IKKα in the noncanonical NF-κB pathway [3]. Although IKKβ and IKKγ are present in these cells, increased p100 may inactivate canonical NF-κB signaling by blocking p65/p50 translocation from the cytoplasm to the nucleus. Thus, IKKα inactivation can impair canonical NF-κB activity through the noncanonical NF-κB pathway in B cells. On the other hand, NF-κB and IKK activities are elevated in *Ikkα^−/−^* keratinocytes compared to WT keratinocytes, following TNF or IL-1 stimulation [24]. Because IKKβ has stronger kinase activity than IKKα, it is speculated that replacing IKKα with IKKβ in the IKK complex in keratinocytes can elevate IKK activity. Interestingly and consistently, we observed increased IKK activity in skin carcinomas lacking IKKα, which was induced by DMBA and TPA [41]. It is not clear whether increased IKK activity is involved in promoting the tumor progression. Thus, regulation of the physiological activities of IKKα, IKK, and NF-κB in different organs remains a mystery. 

Although IKKα deletion in keratinocytes induces spontaneous skin carcinomas, we observe increased IKKα in some human skin SCCs [65,66] (our unpublished data). Immuno-staining reveals that most of the increased IKKα is located in the cytoplasm. Also, we have observed that reintroduced IKKα induces terminal differentiation in primary cultured IKKα-null keratinocytes; however, immortalized IKKα-null keratinocytes are not inducible for terminal differentiation by reintroduced IKKα, suggesting that the program for regulating cell differentiation in the transformed keratinocytes may not be functional or may be altered [33]. Thus, these transformed cells do not respond to normal IKKα signals, whereas increased cytoplasmic IKKα may mimic IKKβ function in tumor cells. Moreover, *Ikkα* mutations have been reported in human skin SCCs [47]. We also isolated IKKα mutations from mouse skin carcinomas, which were induced by DMBA/TPA, and demonstrated that the *Ikkα* mutations can inactivate the IKKα function in inducing keratinocyte terminal differentiation compared to WT IKKα [5]. On the other hand, because we found that overexpressed IKKα in the epidermis inhibited UVB-induced skin carcinogenesis, it is also possible that the distinct pathways may pay a predominant role in driving skin tumorigenesis in those human SCCs that express increased IKKα. Taken together, the new targets and precise functions of IKKα in tumor cells still need to be addressed for clarification of the role of IKKα in tumorigenesis. 
